# Impact of successful local ablative bridging therapy prior to liver transplantation on long-term survival in patients with hepatocellular carcinoma in cirrhosis

**DOI:** 10.1007/s00432-020-03215-9

**Published:** 2020-04-30

**Authors:** Astrid Bauschke, Annelore Altendorf-Hofmann, Michael Ardelt, Herman Kissler, Hans-Michael Tautenhahn, Utz Settmacher

**Affiliations:** Department of General, Visceral and Vascular Surgery, University Hospital Jena, Erlanger Allee 101, 07740 Jena, Germany

**Keywords:** Bridging therapy, HCC, Liver transplantation, Long term survival

## Abstract

**Background:**

It has been shown that local ablative procedures enable downsizing, reduce drop-out from the waiting list and improve prognosis after liver transplantation. It is still unclear whether a response to the local ablative therapy is due to a favorable tumor biology or if a real benefit in tumor stabilization exists, particularly in complete pathological response.

**Method:**

Data of 163 HCC patients who underwent liver transplantation were extracted from our prospectively maintained registry. We analyzed the tumor load, pre-transplant α-fetoprotein levels, child stage aside the application and success of local ablative therapies as bridging procedures before transplantation.

**Results:**

87 patients received multiple and/or combined local therapies. In 20 cases, this resulted in a complete remission of the tumor as observed in the explant histology. The other 76 patients underwent no bridging procedure. The observed 5- and 10-year survival rates for patients with bridging were 67% and 47% and without bridging 56% and 46%, respectively. Tumor-related 10-year survival showed a statistically significant difference between both groups (81% versus 59%). In the multivariate analyses bridging, number of lesions and α-fetoprotein level showed an independent statistically significant influence on tumor-related survival in these patients.

**Conclusions:**

Successful local ablative therapy before liver transplantation is an independent statistically significant factor in long-term tumor-related survival for patients with HCC in cirrhosis and reduces tumor recurrences.

## Introduction

Transarterial chemoembolization (TACE), radio frequency ablation (RFA), radioembolization (RE), percutaneous alcohol injection (PEI), microwave ablation, irreversible electroporation and stereotactic body radiation therapy (SBRT) in combination with thyrosinkinase inhibitor are being employed for local ablative treatment prior to liver transplantation (LT) in selected patients [1–7]. TACE is the most frequently employed bridging procedure. The role of bridging therapy was first reported by Manjo et al. in a study of the Hospital Paul Brousse, Villejuif, France [8]. The procedures are often combined and/or repeated for increased efficiency [9]. It has been shown that local ablative procedures enable downsizing, reduce drop-out from the waiting list and improve prognosis after liver transplantation [5, 10–14]. A statistically significant improvement of survival has been documented in a multi-center study of the European Liver Transplant Registry (Pommergard 2018). It is still unclear whether a response to the local ablative therapy is due to a favorable tumor biology or if a real benefit in tumor stabilization exists, particularly in complete pathological response.

Here, we present the effect of local ablative bridging procedures on the 10-year recurrence rate and the tumor-related 10-year survival after liver transplantation for HCC in our patient population. To the best of our knowledge, this is the first long-term observation in this field.

## Materials and methods

From our prospectively maintained tumor register, we extracted data of HCC patients who underwent liver transplantation between 1996 and 2017. Patients who died within 3 months after LT and patients transplanted for recurrence of HCC after partial hepatectomy were excluded. All patients were followed-up until death or until August, 1st 2018. 163 patients were included in the study.

In cases of sufficient liver function bridging procedures, such as liver resection, local ablative procedures (transarterial chemoembolization (TACE), radio frequency ablation (RFA), Yttrium^90^ radio embolization (Y^90^RE), tomotherapy, in combination with systemic therapy with thyrosinkinase inhibitor were employed since 2004. All these interventions were continued for as long as residual tumor was identified and monitored radiologically in 90 days intervals. In cases of residual vital tumor, the procedures were repeated and combined. TACE was the most frequently applied local ablative therapy. In 87 patients (54%), TACE, RFA or Y^90^ radioembolisation was performed, some of them repeatedly. 67, 13 and 7 cases, respectively, (77%, 15%, und 8%) were TACE, RFA or Y^90^ radioembolisation. 79 patients did not receive any bridging therapy (transplantation before 2004, or functionally unsuitable for local ablative procedures).

133 patients received a deceased donor liver. 30 patients received a split from a living donor (all of them received segments SV-VIII). The waiting time prior to living donation was median 6 months (range 0–20 months) and, thus statistically significantly shorter than prior to deceased donor transplantation (median 9 months, range 0–46). mTOR-based long-term immunosuppression was administered since 2010 in patients who had no contraindications.

Follow-up consultations after LT for HCC are standardized including follow-up for tumor status. As long as laboratory tests including AFP were within normal ranges, a CT scan was performed every 3 months for the first 2 years and then annually. If tumor recurrence was confirmed, therapeutic options were discussed in the interdisciplinary hepatobiliary tumor board. We performed surgical resections with curative intent for intra- and extra-hepatic recurrence, applied local therapy (TACE, Y^90^RE, RFA) in non-resectable intra-hepatic recurrences and radiation for bone metastases. Whenever possible, a systemic therapy with thyrosinkinase inhibitors followed.

We analyzed the morphological data of the tumor load in pre-transplant contrast computed tomography (CT) or magnetic resonance imaging (MRI) scans, α-fetoprotein (AFP) (ng/ml) level, stage of underlying liver disease (Child stage) and use of loco-regional therapy.

All patients gave their consent for clinical registration. We have only used data from the clinical data registry. The study in humans has been carried out with approval of the local ethics committee (Nr. 4337-02/15), in accordance with national law and the Declaration of Helsinki of 1975 (in the current revised form).

The statistical univariate analysis was performed with SPSS Software version 19. Differences in the distribution of variables have been tested with Fisher’s exact test or with Chi square test for statistically significant differences. Survival rates were calculated with the Kaplan–Meier procedure and significance testing was performed with the log-rank test. Starting point for survival was date of transplantation. End point for observed and tumor-related survival were death of any cause and death of hepatocellular carcinoma, respectively. Cox regression analysis was used for the multivariate analysis.

## Results

Patient age at transplantation was median 61 years (range 38–72 years). Morphological tumor load was „inside Milan“ in 70 (43%) patients and „outside Milan“ in 93 (57%) patients. Further data on patients and tumor load are being shown in Table 1. Child- and UICC stage were different in patients with and without bridging. Bridging resulted in complete remission (no evidence of vital tumor tissue in the explant specimen) in 20 cases. This was most frequently observed in single lesions (13/42 = 31% vs. 7/45 = 16%) and after RFA (5/10 vs. 15/77 = 20%), respectively.

**Table 1:** Patients under study

Item	Total	Bridging				*P*
		No		Yes		
		*n*	%	*n*	%	
Total	163	76		87		–
Sex						
Male	135	63	46.7	72	53.3	ns
Female	28	13	46.4	15	53.6	
Age						
< 60 years	81	42	51.9	39	48.1	ns
≥ 60 years	82	34	41.5	48	58.5	
Child stage						
Other	16	11	68.8	5	31.3	0.009
Child A	64	23	35.9	41	64.1	
Child B	55	23	41.8	32	58.2	
Child C	28	19	67.9	9	32.1	
Underlying liver disease						
Other	18	11	61.1	7	38.9	ns
Alcoholic	92	39	42.4	53	57.6	
Hepatitis	41	19	46.3	22	53.7	
Cryptogenic	12	7	58.3	5	41.7	
Number of lesions						
1 lesion	83	42	50.6	41	49.4	ns
2–3 lesions	37	14	37.8	23	62.2	
≥ 4 lesions	43	20	46.5	23	53.5	
Multiplicity						
Solitary	85	43	50.6	42	49.4	ns
Multiple	78	33	42.3	45	57.7	
Diameter of lesions (Maximum)						
< 5 cm	94	41	43.6	53	56.4	ns
≥ 5 cm	69	35	50.7	34	49.3	
α-Fetoprotein (ng/ml)^b^						
< 35 ng/ml (normal)	109	47	43.1	62	56.9	ns
≥ 35 ng/ml (elevated)	46	27	58.7	19	41.3	
< 400 ng/ml	141	66	89.2	75	92.6	ns
≥ 400 ng/ml	14	8	10.8	6	7.4	
Portal vein thrombosis^a^						
No	148	68	45.9	80	54.1	ns
Yes	15	8	53.3	7	46.7	
Extent of hepatic tumor						
Solitary, ≤ 50%	83	41	49.4	42	50.6	ns
Multiple, ≤ 50%	58	23	39.7	35	60.3	
> 50%	22	12	54.5	10	45.5	
Milan						
Milan in	70	33	47.1	37	52.9	ns
Milan out	93	43	46.2	50	53.8	
UCSF						
UCSF in	87	38	50.0	49	56.3	ns
UCSF out	76	38	50.0	38	43.7	
UICC stage^c^						
Stage I/II	100	47	47.0	53	53.0	0.029
Stage III/IV	43	29	67.4	14	32.6	

*ns* no statistically significant difference

^a^No macrovascular invasion

^b^8 missing

^c^20 missing

Median follow-up time after LT was 55 months (range 8–264). By now, 71 patients died, 34 of them of other causes and 37 (22%) of them from HCC recurrence. 5 patients died from malignant second tumor (lung cancer in 3, pancreatic cancer in one and urinary bladder cancer in one), and 29 died from other causes.

Of the 92 living patients, 10 developed a second carcinoma after LT (3 each in the ENT area or on the skin, one each B-cell lymphoma, stomach GIST, small cell renal carcinoma or prostate cancer).

**Fig. 1:**
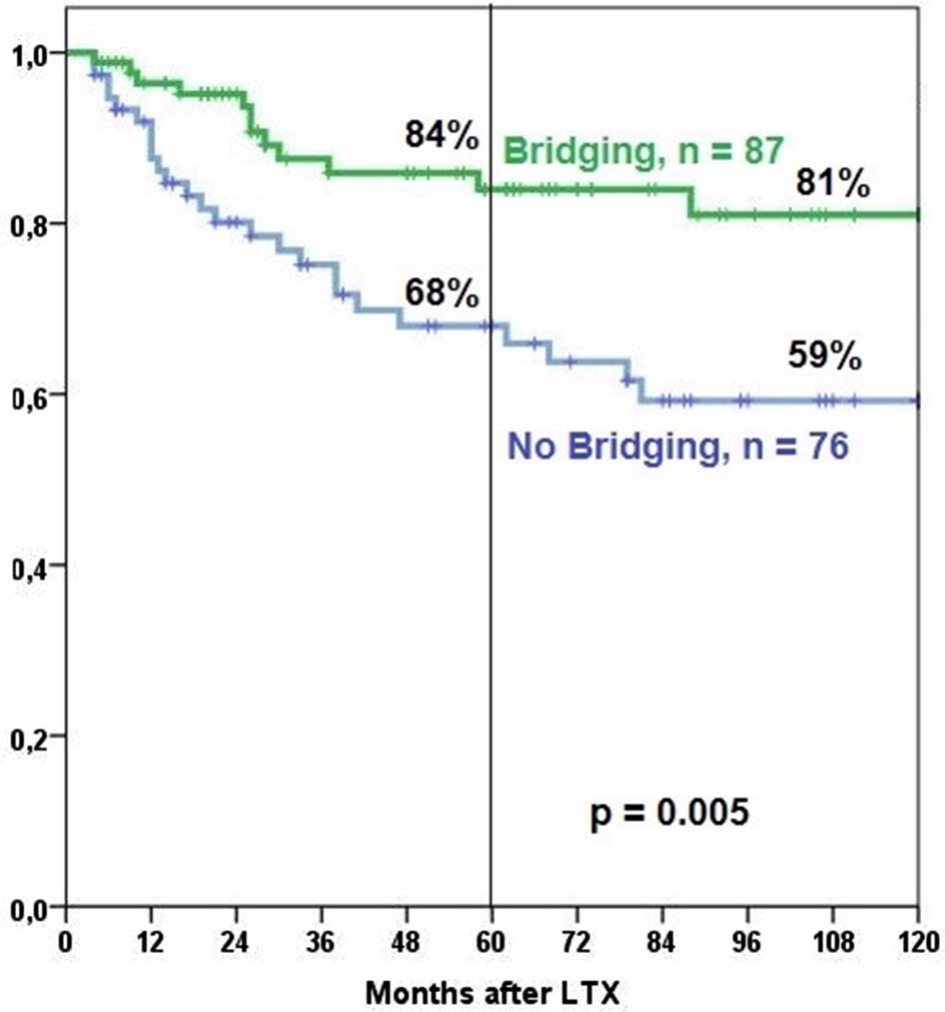
Tumor-related survival of patients with versus without bridging therapy

**Fig. 2:**
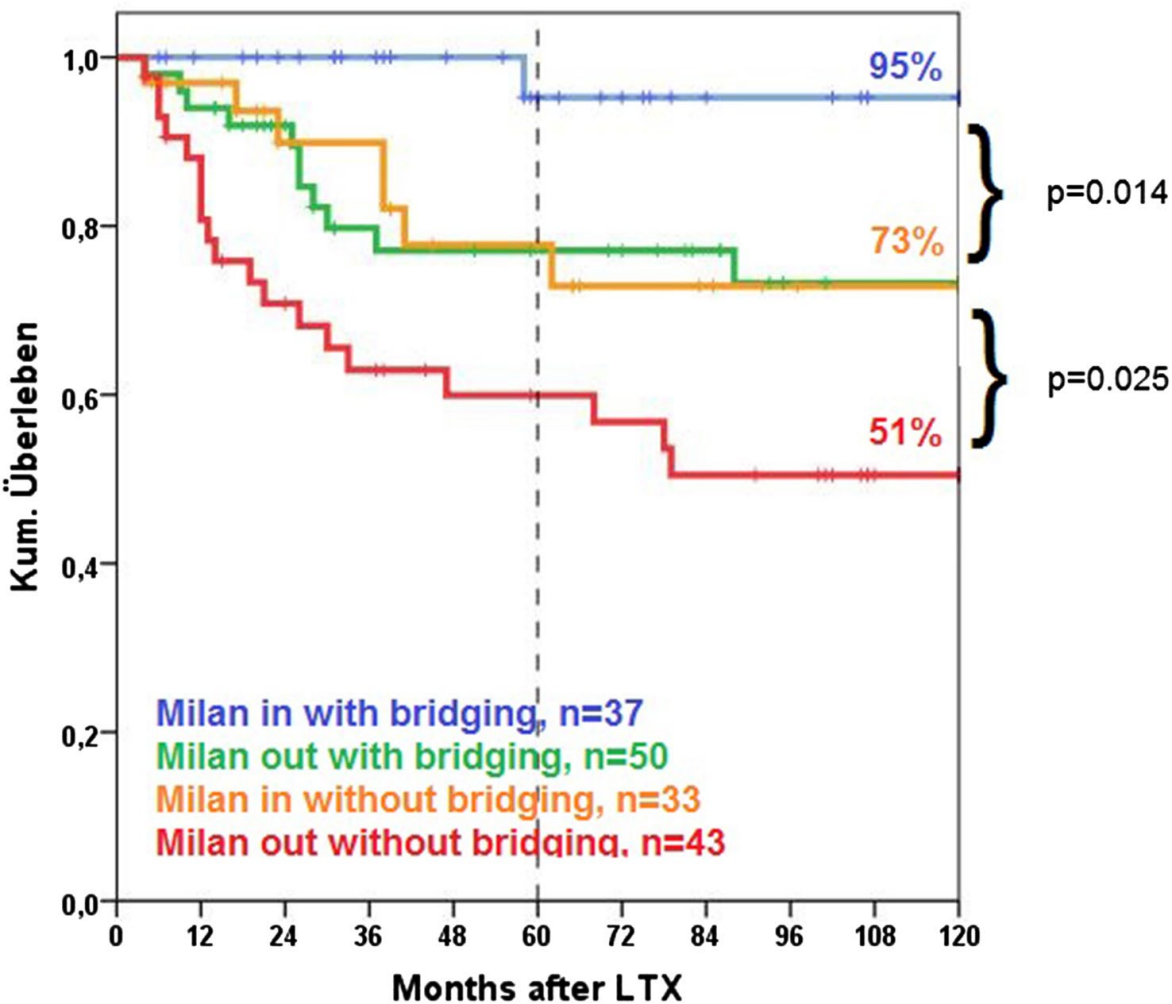
Tumor-related survival of patients with versus without bridging therapy stratified according to Milan criteria

Observed 5- and 10-year survival rates were 62% and 47%, respectively. Observed 5-year survival rate was higher with bridging than without (67% vs. 56%). The difference did not reach statistical significance, though. The respective 10-year survival rates were 47% and 46%, respectively. For tumor-related survival, there was a statistically significant difference in long-term survival (Fig. 1). In addition to bridging, tumor-related survival was statistically significantly related by the level of pre-operative α-fetoprotein levels, Milan and UICC classification (Table 2, Fig. 2).

**Table 2:** Univariat 10-year tumor related survival

Item	n	%	*p*
Total	163	70 ± 4	–
Multiplicity			
Solitary	85	82 ± 5	0.002
Multiple	78	57 ± 7	
α-Fetoprotein (ng/ml)^*1^			
< 35 ng/ml (normal)	109	76 ± 5	0.014
≥ 35 ng/ml (elevated)	46	61 ± 8	
Milan			
Milan in	70	61±6	0.003
Milan out	93	69 ± 5	
UICC stage			
stage I/II	120	81 ± 4	<0.001
stage III/IV	43	44 ± 9	
Bridging			
yes	76	81 ± 5	0.005
no	87	59 ± 7	

^*1^ 8 missing

Sex, patient age, type of bridging procedure, existing portal vein thrombosis and child stage had no statistically significant influence on tumor-related 10-year survival in the univariate analysis.

The statistically significant influence of bridging was maintained with stratification according to Milan classification (Fig. 2).

Of the 20 patients with complete pathological response after bridging, none has died from tumor recurrence so far. 12/20 had been histologically confirmed pre-LT, the others were classified as HCC from CT/MRT scans and AFP.

Bridging therapy, level of pre-operative α-fetoprotein and number of lesions showed an independent statistically significant impact on tumor-free survival in the multivariate analysis (Table 3).

**Table 3:** Multivariate 10-year tumor related survival

	p	Hazard	95,0% confidence interval
Bridging	0.010	2562	1249–5257
Multiplicity	0.001	3207	1575–6531
α-Fetoprotein (ng/ml)	0.007	2519	1284–4944

### Recurrence

46 patients (28%) developed a tumor recurrence. 13 recurrences were located intra-hepatically. Of the 33 extra-hepatic recurrences lung, bones, adrenal glands and peritoneum were affected in 12, 9, 5, 4 patients; abdominal wall, thoracic wall and pericardium were affected in one each.

Following LT with prior bridging, one patient developed an intra-hepatic recurrence, 16 patients (18%) developed extra-hepatic recurrence. Without prior bridging, intra-hepatic recurrence was observed in 12 patients (16%), extra-hepatic recurrence in 17 patients (22%). The rates of intra-hepatic recurrence were statistically significantly different (*p* = 0.001).

Duration of recurrence diagnosis from LT was median 12 months (range 2–62). Where possible, recurrences were treated with curative intent. In cases of intra-hepatic recurrence (IHR), patients survived for median 2 months and in cases of extra-hepatic recurrence (EHR) 18 months. Patients treated with curative intent survived the recurrence for median 38 months, patients treated with palliative intent 6 months.

## Discussion

To our best knowledge, this is the first monocentric series that shows a statistically independent influence of bridging therapy on tumor-related 10-year survival after liver transplantation in patients with HCC in cirrhosis. In patients with bridging therapy, the rate of intra-hepatic recurrence decreased from 16 to 1%, but the rate of extra-hepatic recurrence was unchanged. 20 patients had a complete pathological response. None of them died of recurrence as yet. Only transplanted patients were enrolled in the study. The majority of patients which were removed from the transplant waiting list had distant metastases or intra-hepatic tumor progress. Rarely, patients were removed from the list for other reasons, like aggravation of comorbidities, non-compliance or newly diagnosed second primary tumors. If liver function was sufficient, loco-regional ablative therapy was carried out in these patients. Unfortunately, these data are missing due the retrospective data analysis.

EASL/EORTC guidelines and the international consensus conference for liver transplantation for hepatocellular carcinoma recommend bridging therapy if the waiting time exceeds 6 months [15, 16]. The American guidelines from the AASLD states bridging therapy as the method to prevent tumor progression and drop out from the waiting list [17].

Studies on bridging therapies are extremely heterogenous. There are no recommendations concerning indications for the different procedures, choice of procedure in remaining vital tumor tissue as well as documentation and quality assurance of local ablative therapies. The choice of procedures often depends on local availability. Thus, comparability and reporting the results of these procedures are extremely difficult. In the literature as well as in our study, TACE was the most frequently employed bridging procedure. Currently, there are only small case series on ^90^Y RE as successful bridging therapy prior to transplantation [6, 18–21]. Prospective randomized studies are missing, thus an evaluation of the ^90^Y RE is not possible to date. In selected patients with a maximum of three tumor lesions, each with a maximum diameter of 3 cm, RFA can be performed safely and effectively. Also, the large proportion of complete histological tumor absence in 7/13 patients treated with RFA in our study results from the limited tumor burden.

Primary tumor size and number of lesions vary (Yao und Fidelman 2016; [22]). In our series, transplantation was performed in 93/163 patients with HCC “outside Milan”. The efficiency of bridging therapy in large tumors for meeting the Milan criteria was reported [22, 23]. In our series, too, the effect of bridging in small and large tumor burden was observed.

### Short-term effect of bridging

Numerous studies on bridging prior to LT evaluate the success from imaging studies, employing different standards for remission. Successful radiological criteria of downstaging are tumor shrinkage by 30 or 50% [5, 24], meeting the Milan criteria [6, 25, 26], meeting the Milan criteria with definition of AFP target levels [22, 27], meeting the UCSF criteria [23] as well as complete or partial response according to the mRECIST criteria [28]. The pathological report of the explanted liver for evaluation of response behavior is used only in some studies in addition to imaging studies after bridging. Shaker et al. have shown that radiological response was more pronounced than pathological evaluation of response from the explant specimen [29].

In the literature, information on complete pathological response after bridging therapy ranges from 10 to 54% in different procedures. In our patients, we saw complete pathological response after bridging in 20 of 87 patients (23%) (Table 4).

**Table 4:** Pathological response after bridging therapy

	Time period	Number of Patient with Bridging therapy	Path complete remission	Type of bridging
Mazzaferro et al. (2004)	1998–2003	50	54%	RFA
Mannina et al. (2017)	2008–2015	38	53%	SBRT
El-Gazzaz et al. (2013)	2002–2011	128	39%	DEB-TACE, RE, RFA
Barakat et al. (2010)	2003–2006	14	38%	TACE, RFA, RE
Rubinstein et al. (2017)	2009–2014	50	30%	TACE, RFA, MW, RE
Moore et al. (2017)	2011–2016	23	27%	SBRT
Bargellini et al. (2010)	1997–2006	33	10%	TACE
Radunz et al. (2017)	2007–2015	40	42%	RE
Seehofer et al. (2012)	1989–2008	71	18%	TACE
Agopian et al. (2015)	1994–2013	501	25%	TACE, RFA, PEI
Na et al. (2016)	2003–2012	52	49%	TACE, RFA, PEI
Own data	1996–2017	87	23%	DEB-TACE, RFA, RE, SBRT

*TACE* transarterial chemoembolisation, *DEB-TACE* drug-eluting beads“ transarterial chemoembolisation, *RE* radioembolisation, *RFA* radio frequency ablation, *SBRT* stereotactic Body Radiation Therapy, *MW* microwave ablatio

The efficiency of local ablative therapy has been documented by various radiological studies [30, 31]. Lei et al. reported superior overall survival and tumor-free survival in the group of responders (patients with response (mRECIST) after local ablative therapy with TACE). In particular, the long-term results of patients with HCC outside Milan were after TACE with response better (*p* < 0.05). In the univariate analysis as well as in the multivariate analysis, complete and partial response after TACE was the best predictor for survival and tumor-free survival [32]. Discrepancies of 25% between radiological and pathological response have been reported [33]. Thus, there is an uncertainty to date.

Other factors, such as waiting time for the donor organ or immunosuppression may influence the recurrence rate after liver transplantation [34–36].

### Long-term effect of bridging

Few studies address the impact of bridging therapy on long-term survival. Here, too, different target criteria are being used (overall survival, disease specific survival, recurrence rate). The observed 5-year survival in patients with bridging therapy prior to transplantation for HCC in cirrhosis ranges from 55 to 94% in the literature [25, 37] Only Lee et al. reported 10-year survival of 42% with bridging therapy [38].

The significant impact of tumor number on long-term survival in our series has also been documented by Pawlik et al. and Llovet et al. [39, 40]. Na et al. and Jang et al. reported in agreement with our series a statistically significant impact of AFP levels on long-term survival [37, 41]. Agopian et al. reported a significant impact of AFP on the long-term results in correlation to response to bridging therapy [42]. Comparative studies between patients with and without bridging therapy are listed in Table 5. In the European register study of Pommergraad et al., there was a statistically significant difference in survival of patients transplanted for HCC with versus without bridging therapy [14]. In the other studies, no statistically significant difference was reported. 10-year survival rates have been reported by Seehofer et al. only, according to our knowledge (Table 5).

**Table 5:** Literature references on impact of bridging therapy on long term survival OS overall survival, RFS recurrence free survival, DSS disease specific survival

	Time period	Patients w/o bridging	Median follow up mo.	5 year survival w/o bridging	*p*
Majno et al. (1997)	1985–1995	54/57	40	RFS: 57% / 59%	n.s.
Seehofer et al. (2012)	1989–2008	71/106	_	OS: 73%/ 69%	n.s.
Agopian et al. (2017)*	2002–2013	2754/747	46,7	RFS: 68% / 68%	n.s.
Pommergaard et al. (2018)*	1990–2016	4978/23124	26	OS: 69,7%/ 65,8%	< 0.001
Jena 2019	1996–2017	87/76	55	OS: 67±5% / 56±5% DSS: 84% ±5% / 81% ±5%	n.s.

*OS* overall survival, *RFS* recurrence free survival, *DSS* disease specific survival

*multicentric

### Summary

To reduce prognostically relevant recurrence after LT different options are being investigated. In our series, response behavior to bridging therapy prior to transplantation for HCC in the explanted liver had a positive impact on long-term survival, cumulative tumor recurrence rate and location of tumor recurrence after LT.

### Lay summary

We report on a single-center analysis of all local ablative bridging procedures (TACE, RFA, RE, PEI) prior to liver transplantation with 10-year long-term survival.
